# MetaGeneHunt for protein domain annotation in short-read metagenomes

**DOI:** 10.1038/s41598-020-63775-1

**Published:** 2020-05-07

**Authors:** R. Berlemont, N. Winans, D. Talamantes, H. Dang, H-W. Tsai

**Affiliations:** 1 Department of biological Sciences, California State University, Long Beach, California, USA; 2 Present Address: Department of Bioinformatics, University of Georgia Athens, Athens, Georgia USA

**Keywords:** Functional clustering, Microbial communities

## Abstract

The annotation of short-reads metagenomes is an essential process to understand the functional potential of sequenced microbial communities. Annotation techniques based solely on the identification of local matches tend to confound local sequence similarity and overall protein homology and thus don’t mirror the complex multidomain architecture and the shuffling of functional domains in many protein families. Here, we present MetaGeneHunt to identify specific protein domains and to normalize the hit-counts based on the domain length. We used MetaGeneHunt to investigate the potential for carbohydrate processing in the mouse gastrointestinal tract. We sampled, sequenced, and analyzed the microbial communities associated with the bolus in the stomach, intestine, cecum, and colon of five captive mice. Focusing on Glycoside Hydrolases (GHs) we found that, across samples, 58.3% of the 4,726,023 short-read sequences matching with a GH domain-containing protein were located outside the domain of interest. Next, before comparing the samples, the counts of localized hits matching the domains of interest were normalized to account for the corresponding domain length. Microbial communities in the intestine and cecum displayed characteristic GH profiles matching distinct microbial assemblages. Conversely, the stomach and colon were associated with structurally and functionally more diverse and variable microbial communities. Across samples, despite fluctuations, changes in the functional potential for carbohydrate processing correlated with changes in community composition. Overall MetaGeneHunt is a new way to quickly and precisely identify discrete protein domains in sequenced metagenomes processed with MG-RAST. In addition, using the sister program “GeneHunt” to create custom Reference Annotation Table, MetaGeneHunt provides an unprecedented way to (re)investigate the precise distribution of any protein domain in short-reads metagenomes.

## Introduction

Over the past decades, high throughput DNA sequencing of metagenomic DNA has generated a huge amount of sequences whose characterization has provided unprecedented insights into the structure and function of microbiomes^1–4^. Although metagenomes have been assembled^5–7^, the vast majority of published metagenomic data still consists of unassembled short-read DNA sequences. For example ~94% of the ~15,000 datasets listed in the TerrestrialMetagenomeDB are short-read metagenomes^8^. The annotation of these short-reads generally involved the identification of local similarities with sequences in a pre-annotated reference database (e.g., M5nr)^3,9^ or specialized database (e.g., CAZy)^1,2^. Generally, the processing of these short-reads requires specific computational approaches and infrastructures^5^ however the development of publicly accessible annotation platforms (e.g., MG-RAST, IMG)^9,10^, sometimes used for repository, has somewhat addressed the computational challenge^5^. For example, as of December 2019, MG-RAST hosted ~400,000 publicly accessible annotated datasets^9^. Nevertheless, although short-reads annotation enables one to investigate communities’ structure and functional potential (i.e., the predicted function of the microbiome based on sequence annotation), this approach has multiple caveats^5^. Among others, identifying local sequence similarity between short-read queries and target sequences in database does not guarantee global sequence similarity/homology over the entire sequence. Thus, confounding local matches with global/functional similarity introduces biases as many proteins consist of complex and variable assemblages of distinct functional protein domains^11–13^. In this context, a domain-centric annotation system is needed to discriminate the catalytic domain supporting the function of interest from their accessory domains and thus to better investigate the functional potential of sequenced microbial communities. However, to date, the direct identification of protein domains (e.g., HMM profiles) in short-reads datasets is not reliable and poorly developed^5^.

Identifying the domains, rather than the proteins, will also allow for the normalization of the hit to account for the domain length. Indeed, the proportion of short-reads matching a domain of interest (or an entire protein) is affected by the length of the domain as the raw number of sequences matching specific domains is expected to reflect both the abundance and the length of the domains. Not accounting for the length of the domain (or protein) of interest during the data processing introduces two major systematic biases. First, the longer the domain of interest; the more their frequency is overestimated. Second, if the data processing involves rarefaction, short, less abundant domains, although important, might be discarded.

To address these problems, we designed MetaGeneHunt to perform precise annotation of protein domains in short-reads metagenomes retrieved from MG-RAST. MetaGeneHunt combines short-read local alignment, provided by MG-RAST, with precise PFam-based^14^ protein domain identification in the M5nr database^15^ to identify protein domains in publicly accessible datasets. Here, we focused on microbial glycoside hydrolases (GHs) as these enzymes are essential^16^ for the processing of carbohydrates in mammals’ gut^1,2^ and across environments^3^ where they support the carbon cycling. Like other carbohydrate active enzymes (CAZymes) and many other protein families, GHs are known for their complex multidomain architecture^15^. Although most biochemically characterized GH-enzymes consist of single-domain GHs (SDGHs), the bioinformatic analysis of sequenced genomes and assembled metagenomes has highlighted the complexity and abundance of multi-domain GHs (MDGHs)^12,15,17^. For example, ~60% of identified α-amylases from GH13 contain multiple domains. Similarly up to ~25% of known enzymes active on cellulose, xylan, and chitin are MDGHs^15^. In addition, many “non-CAZy” domains, including some α/β-hydrolases and many domains of unknown function (DUFs), are linked to CAZy domains. Focusing on local alignment to identify the functional potential of sequenced microbial communities [e.g.,^1–3^] overestimate the frequency of the trait of interest whereas identifying matches between short-reads and GH-domains (e.g., GH13), GH sub-domains (e.g., GH30C), accessory domains (e.g., CBM2), or a DUF should only count toward this specific domain.

We used MetaGeneHunt on publicly accessible metagenomes from MG-RAST including the Mammal Microbiome^1^ and the Twin Gut Microflora study^2^, to evaluate the frequency of short-reads matching with GH-containing protein and the frequency of matches located in the GH domains. We next used MetaGeneHunt to investigate how the functional potential for carbohydrate processing in microbial communities associated with food fluctuates as the bolus moved through the gastrointestinal tract (GIT) of five captive mice (*Mus musculus*). We sampled the bolus along the GIT, rather than feces, because the GIT consists of a series of interconnected environments hosting distinct microbial communities supporting specific processes^18–21^. Although distinct microbial communities inhabit the mucosal and luminal space in the GIT^19,22,23^, we decided to investigate the precise distribution of GHs, and their associated domains, in microbial communities associated with the bolus (in the lumen) as these communities interact directly with the substrate. More precisely, we investigated communities in the stomach, intestine, cecum, and colon.We hypothesized that the community structure along the GIT will be similar for all the mice and that the sampled sections of the GIT will be associated with distinct conserved microbial communities across individuals. Indeed, these healthy mice shared the same genetic background, were maintained in the same conditions, and were fed *ad libitum* with the same plant-based diet. In addition, the sampled sections of the GIT corresponded to anatomically discrete regions, separated from each other by the cardiac, the pyloric, and the ileocecal sphincters, all sections where the bolus is incubated for prolonged time^24^. In these regions, the bolus is exposed to various pH, salts, and enzymes, among other conditions^21,24–27^. These conditions support the host’s endogenous digestive process and produce discrete environments where microbial communities develop and contribute to the overall digestion process by releasing additional enzymes.

As GHs are not evenly distributed across microbial lineages^12,28^, we predicted that changes in microbial community composition along the GIT would correlate with variation in the potential for carbohydrate processing based on identified sequences for GHs. Alternatively, as polysaccharide deconstruction is an essential process supported by multiple lineages^28^, one could hypothesize that structurally distinct communities would converge to similar functional potential^3^. In this case the functional potential would be more conserved than the microbial community structure. Finally, we investigated the correlation between structure and functional potential for carbohydrate processing across microbial communities derived from the same location.

Combining a method for the rapid domain-specific annotation of short-reads datasets with the possibility to (re)analyze publicly accessible from MG-RAST provides an unprecedented opportunity to precisely investigate the functional potential within and across sequenced microbial communities.

## Results

### Glycoside hydrolases identification

We first used MetaGeneHunt on publicly accessible datasets from the Mammal Microbiome^1^ and the Twin Gut Microflora^2^ studies and found that 43.8 and 40.4% of the short-read sequences matching with a GH domain-containing sequence were localized in GH domains, respectively. Next, among the 4,726,023 short-read sequences matching with a GH domain-containing sequence identified in the Mouse GIT (this study), only 1,973,805(41.7%) of the hits were actually localized in a domain of interest (Supplementary Table 1). MetaGeneHunt identified many short-reads matching with GHs, carbohydrate binding modules (CBMs), non-GH CAZy domains such as Glycosyl-Transferase (GT) family 2, phosphorylases, lipases, transporters (e.g., PTS subunits), and many domains of unknown function (DUFs)(Supplementary Table 1).

Next, although GHs were the most abundant domain of interest identified in the mouse GIT, the numbers of hits per GH family were not evenly distributed. Across samples, the total number of GH and CBM domains hits correlated with the size of the domain expressed in amino acids (P_Pearson_ = 0.028, P_Spearman_ < 0.01), thus suggesting that unnormalized raw hit counts systematically overestimated the frequency of the longer domains of interest.

**Figure 1:**
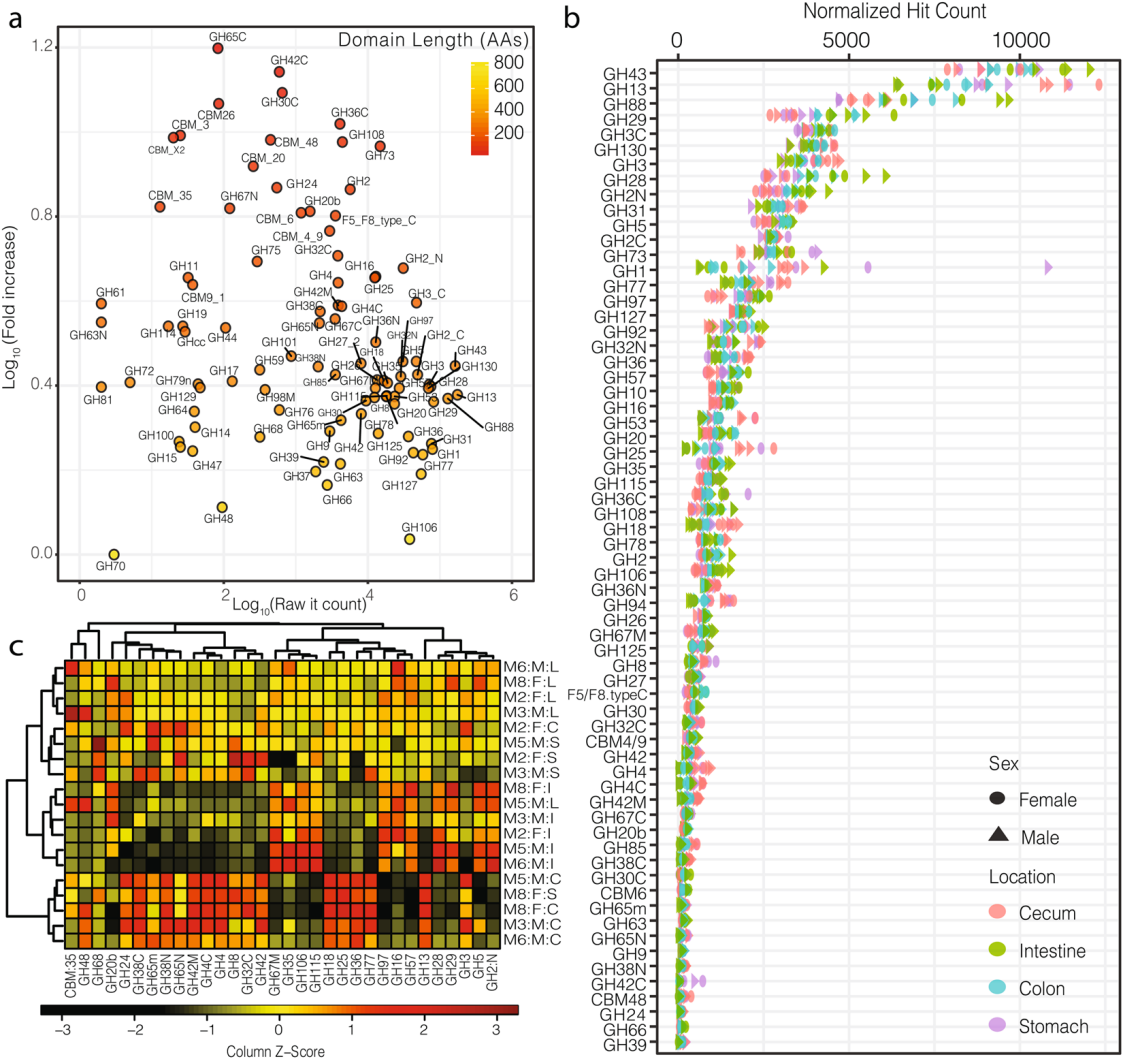
(**a**) Fold increase (log10) in the total normalized domain-specific hit count, accounting for the domain length, relative to the total raw domain specific hit count (log10) in the mouse GIT. (**b**) Rarefied-normalized domain specific hit count across the mouse GIT (only showing domain >100 hits). (**c**) Heatmap showing the distribution of rarefied-normalized GH domains most affected by the sample origin in the mouse GIT (see text, Mx:F/M:S/I/C/L – Mouse#: Female/Male: Stomach/Intestine/Cecum/Colon).

Therefore, in subsequent analyses, localized-hit counts for GHs and CBMs were multiplied by the ratio of the longest identified domain, in this case GH70 (805 AAs), divided by their length (Fig. 1A, Supplementary Tables 1, 2). This normalization mostly affected the short domains (e.g., GH78, GH25), the small subdomains (e.g., GH31N, GH36C), and the accessory domains of interest (e.g., CMB5_12) (Fig. 1A). As expected, the resulting normalized hit count was independent from the domain length (P_Pearson_ = 0.38, P_Spearman_ = 0.33) and thus better mirrored the distribution of the functional domains in the GIT. Next, normalized and localized hit counts were rarefied (n = 99,922)(Fig. 1B, Supplementary Table 2). Sequences for the β-xylosidases/α-L-arabinofuranosidases from GH43 (9.76% of the hits), the α-amylases from GH13 (8.87%), the β-glucuronyl hydrolases from GH88 (6.54%), and the α-L-fucosidase from GH29(4.11%), were the most abundant GHs identified (Supplementary Table 2).

Regarding enzymes targeting structural polysaccharides, domains from GH5, GH8, and GH9, accounting for 2.89, 0.55, and 0.12% of the normalized hits respectively, were the most abundant cellulase domains. No fungal cellulase from GH7, nor GH6, GH44, GH45 and GH48 were detected. We identified sequences encoding potential xylanases from GH10 (~1.3%), GH30 (~0.4%), and few xylanases from GH11. Chitinases from GH18 accounted for 0.91% of the identified sequences whereas a few GH19 were identified but only in some samples. Carbohydrate binding modules (CBMs), together accounting for ~0.7% of the normalized hits, were from the CBM families 48, 4_9, 6, 26, 20, 9_1, and 35 (Supplementary Table 2).

Finally, considering GH families associated with several subdomains, in most cases the frequencies of the individual subdomains matched. For example, regarding the abundant β-glucosidases from GH3, the overall frequency of the GH3 (3.79% of the hits) matched the frequency of the domain GH3C (4.00%). Similarly, regarding the GH2 β-galactosidases, GH2N and GH2C accounted for 3.17 and 2.78% of the hits, respectively (Fig. 1, Supplementary Table 2). Finally, regarding the less abundant GH4, encoding some potential maltose-6-phosphate glucosidases and α-galactosidases, the GH4 and GH4Cdomains accounted for 0.37 and 0.36% of the hits, respectively.

Together this suggested that identifying the protein domains and accounting for their length produces a robust functional annotation system and that the localized/normalized hit counts reflect the actual distribution of the domain of interest.

### Glycoside hydrolases distribution

The distribution of many domains of interest, across samples, was affected by the origin of the sample in the GIT (two-way ANOVAs, GH_Norm_~ Location × Sex), with P-values < 0.001 for many domains including GH8 (cellulase/chitosanase), GH42 (β-galactosidases), GH68 (levansucrases), GH18 (chitinases), GH38 (α-mannosidases), and GH25 (lysozyme) among others (Supplementary Table 3).The distribution of some CBMs including CBM48, CBM3, and CBM9_1 was also affected by the sample origin. Conversely, the distribution of several domains, including GH1, GH3, GH16, GH30, GH31, GH44, CBM4_9, and CBM_2 was not affected by the sample origin and together these traits formed a core set of functional traits across the microbial communities in the mouse GIT.

**Figure 2:**
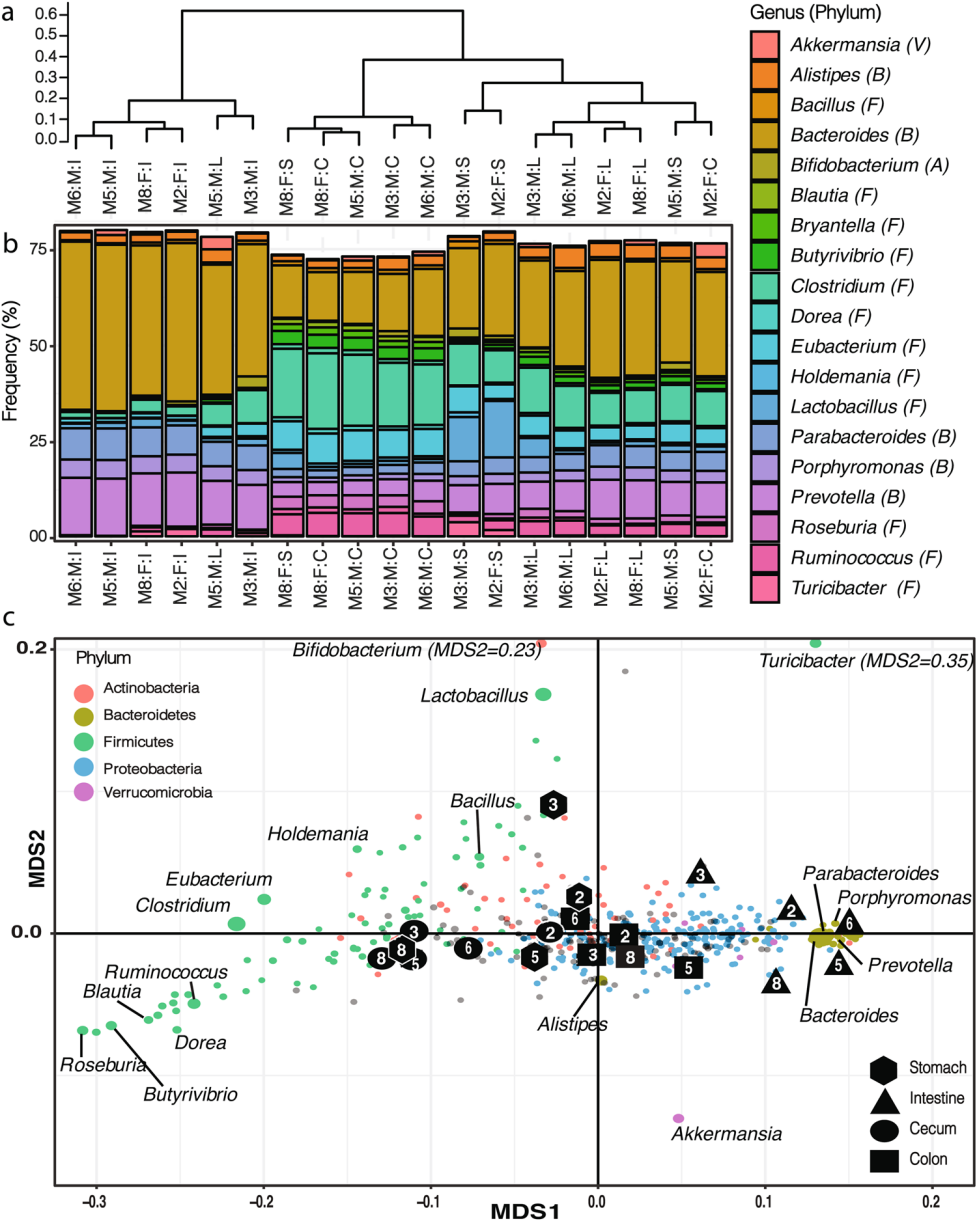
(**a**) Sample clustering based on the (complete) microbial community composition identified at the genus level, after rarefaction, using Bray-Curtis dissimilarity index, and complete linkage. (Mx:F/M:S/I/C/L – Mouse#: Female/Male: Stomach/Intestine/Cecum/Colon). (**b**) Bar-plot highlighting the microbial community composition across samples, for clarity only the genera accounting for at least 1% of community, after rarefaction, of the annotated reads are displayed (V = phylum *Verrucomicrobia*, B = *Bacteroidetes*, A = *Actinobacteria*, F = *Firmicutes*). (**c**) NMDS analysis (2D stress=0.020) revealing the sample clustering overlaid with all the identified bacterial genera. The genera are color-coded by phylum and the major groups, highlighted in (**b**), are labelled individually. The size mirrors the maximum frequency of the genus across samples.

Next, we investigated the samples clustering using the traits most affected by the sample origin (i.e., P < 0.01, Fig. 2B). First, relative to the other origins, samples from the stomach (S) were not enriched in any GHs, relative to the other locations, and thus formed a poorly resolved cluster related to samples from the colon (L) (see below). Next, samples from the intestine (I) formed a more homogeneous and deeply branched cluster enriched in sequences from the abundant GH2 family. Most of the biochemically-characterized members of this GH family encode putative β-galactosidases^15,29^. Similarly, sequences encoding GH5 (cellulases), GH28 (polygalacturonases), GH29 (α-L-fucosidases), and GH97 (α-galactosidases) were more characteristic of the intestine. In addition, some less abundant sequences from GH67 (α-glucuronidases), GH106 (α-L-rhamnosidases), GH26 (β-mannanases), and GH35 (β-galactosidases) were also systematically enriched in the intestine. Conversely, compared to other locations, the intestine contained fewer sequences encoding members of GH13 (α-amylases), GH73 (peptidoglycan hydrolases), GH4 (maltose-6-phosphate glucosidases), GH8 (cellulases), and GH18 (chitinases). Next, most samples from the cecum (C) clustered together and were enriched in many GH families underrepresented in the intestine and including GH13, GH73, GH4, GH8, and GH18 among others. Finally, most samples from the large intestine (L), like samples from the stomach, formed a poorly resolved cluster associated with no specifically enriched GHs.

Thus, beside a set of core GH domains, the distribution of functional traits supporting the polysaccharide deconstruction associated with the bolus is GIT-section specific. This suggested that when transiting from the stomach to the colon, the bolus is exposed to microbial communities associated with distinct functional potential for polysaccharide deconstruction.

### Structure of the mouse gut microbiome

The distribution of taxonomically-annotated sequences, from the phylum to the genus level (Supplementary Fig. 2), was used to estimate the microbial community structure in the bolus as it transited from the stomach (S) to the intestine (I), the cecum (C), and then the colon (L). After rarefaction, all the microbial communities were dominated by members of the *Bacteroidetes* and *Firmicutes* phyla (Fig. 2, S2) and displayed α-diversity (Shannon), computed at the genus level, ranging from 2.7 to 3.5 (Supplementary Fig. 3).

**Figure 3:**
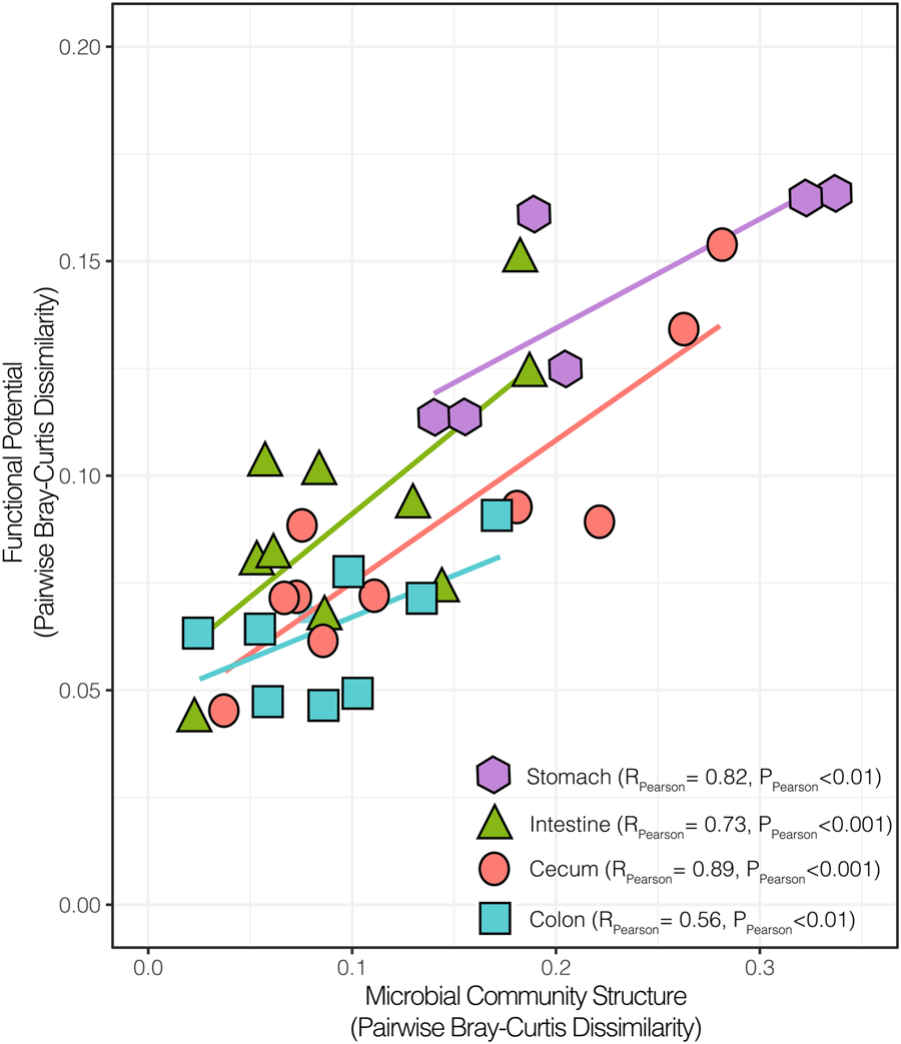
Pairwise comparison of the structural and functional dissimilarities (Bray-Curtis) across samples from the same location, the lines depict the linear regressions.

Samples in the stomach and cecum displayed the highest α-diversity. In addition, the corresponding inferred communities were the most variable across individuals (Figs. 2, 3, S2, S3). Next, following the bolus along the gut, the microbial community in the intestine was less diverse and more conserved across individuals. This microbial assemblage was characterized by abundant *Bacteroidetes *(e.g., *Bacteroides, Parabacteroides*, *Prevotella*) and less abundant lineages from the *Proteobacteria *phylum (Fig. 2, S2), among others. As the bolus proceeded to the cecum, between the intestine and colon, a new conserved and highly diverse community emerged and *Firmicutes* (e.g., *Ruminococcus, Clostridium, Butyvibrio*) and some *Actinobacteria* (e.g., *Bifidobacterium*) became more characteristic (Fig. 2, S2). Finally, when the bolus reached the colon, from the intestine or the cecum, a final community established. This assemblage contained lineages found in the intestine and in the cecum and some less abundant lineages from the phylum *Proteobacteria *(Fig. 2). At large, the community in the colon was more similar to the community in the intestine than the community in the cecum (Supplementary Fig. 3). Finally, communities from the colon and the stomach displayed high level of similarity thus supporting the transfer of material from the colon to the stomach thru coprophagy (Supplementary Fig. 3).

Overall, the sample origin explained 79.6% of the observed variance in the distribution of taxonomically annotated sequences, at the genus level, across samples (PERMANOVA, genus~origin×sex, with 999 permutations) whereas the sex, the individual, and their interaction had no significant effect.

### Structure-function relation in the microbiome

Within GIT locations (i.e., stomach, intestine, cecum, and colon), variation of the community structure, identified at the genus-level, correlated with change of the functional potential for polysaccharide deconstruction with all the P-values below 0.001 except for samples from the large intestine (P_Pearson_ = 0.02) (Fig. 3). Communities from the stomach and the cecum were the most structurally and functionally diverse although diversity remained highly correlated to functional potential. Next, community composition and function were mostly conserved in the intestine whereas the large intestine associated with a conserved microbial community displayed a variable functional potential. (Fig. 3).

## Discussion

MetaGeneHuntis a new way to perform domain specific identification of GHs, and associated domains, in short-read metagenomes. Identifying domains, rather than protein, is essential as GH domains are associated with many variable domains^12,15,30^. This new approach is based on, and complements, the GeneHunt annotation approach^15^ and is designed to analyze short-read metagenomes from MG-RAST^9^. As such, it doesn’t require large computer infrastructure.

According to the proposed approach, identifying domains (i.e. GHs) rather than entire proteins addresses two major problems: (i) it discriminates local matches inside or outside the functional domains of interest, (ii) it allows the normalization of the raw hit count by accounting for the domain length which directly affects the likelihood of matching hits. Systematically considering the local hits in protein containing GH domains and not accounting for the domain length together result in a systematic bias towards the longer domains and more complex proteins (with multiple domains). When investigating the functional potential and the clustering of samples derived from the same environment, this bias is minimized, and the sample clustering remains essentially unchanged, as the same systematic bias is applied repeatedly. However across environments, as the distribution of GH domains is highly variable^3,31^ and as the protein modularity varies^12,15,32,33^ bypassing the normalization results in unknown bias. Although MetaGeneHuntcan identify local hits matching with accessory domains (e.g., lipases), it does provide a comprehensive identification only for the domains listed in the reference annotation table (RAT). In order to further investigate the distribution of these newly identified accessory domains, not targeted at first, a new RAT should be constructed using GeneHunt^15^.

In the mouse GIT, as the bolus transits from the stomach to the colon, it exhibits a series of structurally and functionally distinct microbial communities. Although microbes associated with the bolus come directly with the food or from the preceding sections of the GIT along with the bolus and transfer between the microbial community associated with the mucosa, even sequential parts of the GIT (e.g., intestine and colon) display significantly distinct microbial communities. This suggests that the distinct physiology of sequential section of the GIT cause abrupt modification of the microbial community composition. Being in contact with bolus, these microbial lineages directly contribute to its degradation by releasing enzymes into the lumen^34–36^. In herbivores, the GIT microbiome is enriched in carbohydrate active enzyme targeting cellulose and the hemicellulose, among others^1,37,38^. This plant material represents a major source of carbon and energy for the host and its associated microbial communities. In the mouse, beside a set of core traits not affected by the sample origin, the distribution of several GH families mirrors the GIT compartmentalization. This reflects the broad distribution of traits involved in the processing of short oligosaccharide (e.g., GH1, GH3) in sequenced microbial lineages, whereas traits targeting more complex structural substrates tend to be restricted to specific lineages^12,28,39^.

In the stomach of mice, although initially perceived as a mostly sterile environment due to the low pH^40^, the bolus is associated with the most diverse DNA. As the stomach is the first section of the GIT where the bolus is incubated^24^, the corresponding inferred microbial community likely reflects both the active microbes in the stomach and the DNA in and on the food. Regarding the functional potential for polysaccharide deconstruction, although containing many GH sequences, the stomach is not specifically enriched in any GH family. In addition to core traits, the stomach, like the other section of the GIT, contained a high proportion of sequences encoding amylases from GH13 and GH57, cellulases from GH5, GH8, and GH9, and many enzymes targeting short oligosaccharides (e.g., GH2, GH3, GH31, GH43). Together this suggests that, although enriched in enzymes for carbohydrate processing, there is no selection for specific enzymes in the stomach, relative to the other sections of the GIT.

In the intestine, the somewhat variable bolus-associated community coming from the stomach is filtered and a more conserved microbial community emerges. Across individuals, this community is characterized by the high frequency of Bacteroidetes. More specifically, *Bacteroidaceae*, *Prevotellaceae*, and *Porphyromonadaceae* dominate and account for more than 70% of the reads in the intestine. The systematic investigation of sequenced Bacteroidetes lineages reveals their increased potential for polysaccharide processing^12,41^. Indeed, at large Bacteroidetes are enriched in enzymes targeting short oligosaccharides (e.g., GH2,GH3), starch (GH13), cellulose (mostly GH5), xylan (GH10,GH30), and other polysaccharides (e.g., GH16,GH28, GH29 GH43, GH67, GH97)^12,28,39^. Consistently, when focusing on the normalized distribution of identified GHs, the bolus in the intestine, the high frequency of GH2, GH5, GH28, GH29, GH97, GH67, GH106, GH26, GH35, and GH57 among others, was identified.

Next, from the intestine, some of the bolus goes directly to the colon whereas some goes to the cecum before being transferred to the colon. To date, the exact function of the cecum isn’t fully understood although it might be a reservoir of bacteria to colonize the colon. Also the cecum is the primary site of colonic fiber fermentation supporting the release of short chain fatty acids (SCFA) absorbed in the colon^20,42^. As identified here, the microbial community in the cecum, is dominated by lineages of *Butyvibrio*, *Ruminococcus*, and *Clostridium*, all taxa known or predicted to have high potential for polysaccharide deconstruction^12^. Sequenced genomes from these lineages of *Firmicutes *are enriched in the GH domains identified independently in the short-read metagenome from the cecum. Finally, in the colon, the microbial community is characterized by a mix of microbial lineages with no characteristic taxon, as depicted for the stomach. Accordingly, the GH content, although abundant was not specifically enriched in any function. Interestingly, communities from the colon and stomach, although being the most distal communities characterized here, displayed a high degree of structural and functional similarity. This supported the murine practice of coprophagy where material released from the colon get re-ingested as a way to retain bacterial proteins^43^, to further process some partially digested material^44^, and to re-colonize the GIT^45,46^.

In conclusion, over the past decades, the development of metagenomics and the associated bioinformatics techniques has profoundly affected our understanding of the microbial diversity and its contribution to environmental processes. The functional annotation of short-reads is an essential aspect of this metagenomic revolution. However, a metagenome annotation can only be as good as the annotation of the reference database. Hence, as demonstrated here, the careful domain specific annotation of the M5nr database^15^, combined with the power and convenience of MG-RAST^9^, provides an unprecedented way to quickly and precisely annotate newly sequenced short-readsmetagenomes and to reanalyze publicly accessible datasets [e.g.,^1,2^]. Finally, although the GeneHunt^15^ and MetaGeneHunt approaches were designed for the precise identification of GHs in sequenced genomes and metagenomes, many other proteins such as receptors, display complex and variable multidomain architectures. In the future, the creation of new dedicated RAT using GeneHunt combined with MetaGeneHunt will provide new efficient ways to investigate the functional potential of sequenced metagenomes.

## Methods

### MetaGeneHunt

MetaGeneHunt was designed to work with MG-RAST annotated datasets^9^ (Supplementary Fig. 1). MetaGeneHunt uses a precise domain-specific (PFam^14^) annotation of the Glycoside Hydrolases and accessory domains (e.g., CBMs) in the M5nr database^47^ created using GeneHunt^15^ as a reference annotation table (RAT). First, MetaGeneHunt retrieves the M5nr annotated metagenomes from MG-RAST (i.e., the “330” and “650” files) using the MG-RAST APIs^48^. Next, sequences matching potential GHs are identified in file “650”, using the MD5id of the annotated hits from the RAT. Next, for these local matches, the precise alignment position is compared to the domain-specific annotation in the RAT. If >20AAs from the query align with a specific protein domain (considering the HMM-envelope position in the RAT)^14,49^ then this domain annotation is transferred to the query. Conversely, the annotation is considered negative if >20AAs of the query match outside the domain of interest (e.g., in linker, accessory domain, signal peptide). The cutoff for the overlapping can be modified at will by the user. Next, the actual sequence count for each identified hit is retrieved from the sequence agglomeration file (i.e., file “330”). Finally, in the subsequent data processing and normalization, hit counts, per protein domain, are normalized according to the size of the protein domain in the Pfam database^14^.

### Animal care and use

Samples of bolus were collected from 42-day old, male (n = 3) and female (n = 2), C57BL/6 J mice (Jackson Laboratory, Bar Harbor, ME) housed in the California State University Long Beach Animal Care Facility. Mice were maintained under 12h:12h light:dark cycle and fed *ad libitum* with fiber rich diet (Teklad LM-485, Harlan, Madison, WI). Individuals were sacrificed via rapid decapitation (IACUC #349), then during dissection each region of the gut (i.e., the stomach, the intestine, the cecum, and the large intestine) was sutured in the abdomen to ensure the bolus remained in place. Then the regions were opened aseptically, and the bolus was collected. All experimental procedures were approved by CSULB IACUC and performed according to AAALAC guidelines.

### DNA extraction and sequencing

Total DNA was extracted using PowerFecal DNA Isolation Kit (MoBio, Carlsbad, CA), sheared using a focused ultrasonicator (Covaris, Woburn, MA), tagged using Multiplex TruSeq DNA Nano (Illumina, Carlsbad CA), QCed on Agilent Bioanalyzer, and sequenced on Illumina HiSeq.2500 (PE100) at the UCI Genomics High-Throughput Facility (University of California Irvine, Irvine, CA). DNA sequences were uploaded to MG-RAST for repository and taxonomic annotation^9^.

## Supplementary information

Supplementary Figure 1.

Supplementary Figure 2.

Supplementary Figure 3.

Supplementary Table 1.

Supplementary Table 2.

Supplementary Table 3.

## Data Availability

Raw and preprocessed data used in this manuscript are publicly accessible on the MG-RAST server^9^. The mouse microbiome data, corresponding to ~555 millions of 100 bp sequences, are available in the mgp20861 project. Additional datasets for the Mammal Microbiome data (mgp116)^1^ and the Twin Gut Microflora study (mgp10)^2^ were retrieved using the MG-RAST API^48^. An additional annotation table for glycoside hydrolases (GHs), and related enzymes, in the Mammal Microbiome study^1^ was obtained from Brian Muegge (direct correspondence). Pre-processed data including the taxonomic annotations of the reads, from phylum to genus levels, were retrieved using MG-RAST API^48^. Data analytics and statistics were carried out using R statistical language^50^. MetaGeneHunt and the RAT for GH are publicly accessible on GitHub (https://github.com/renober/MetaGeneHunt).
