# Supplementary figures and images for: MetaGeneHunt for protein domain annotation in short-read metagenomes
Source: Sci Rep. 2020 May 7;10:7712. doi: 10.1038/s41598-020-63775-1 (PMC7205989; doi:10.1038/s41598-020-63775-1)

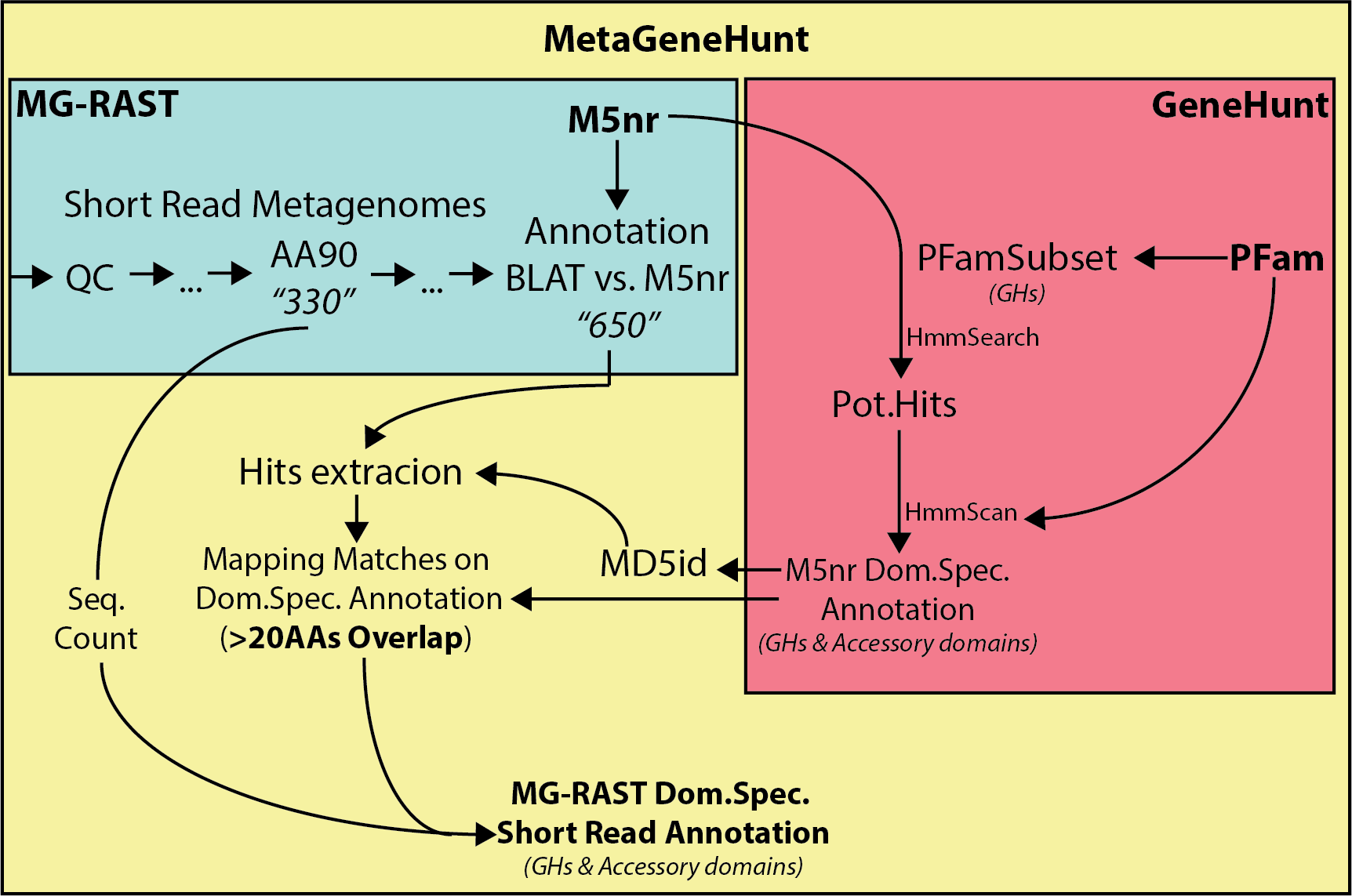

Supplement: Supplementary file 1 — Supplementary Figure 1. [file 41598_2020_63775_MOESM1_ESM.png]

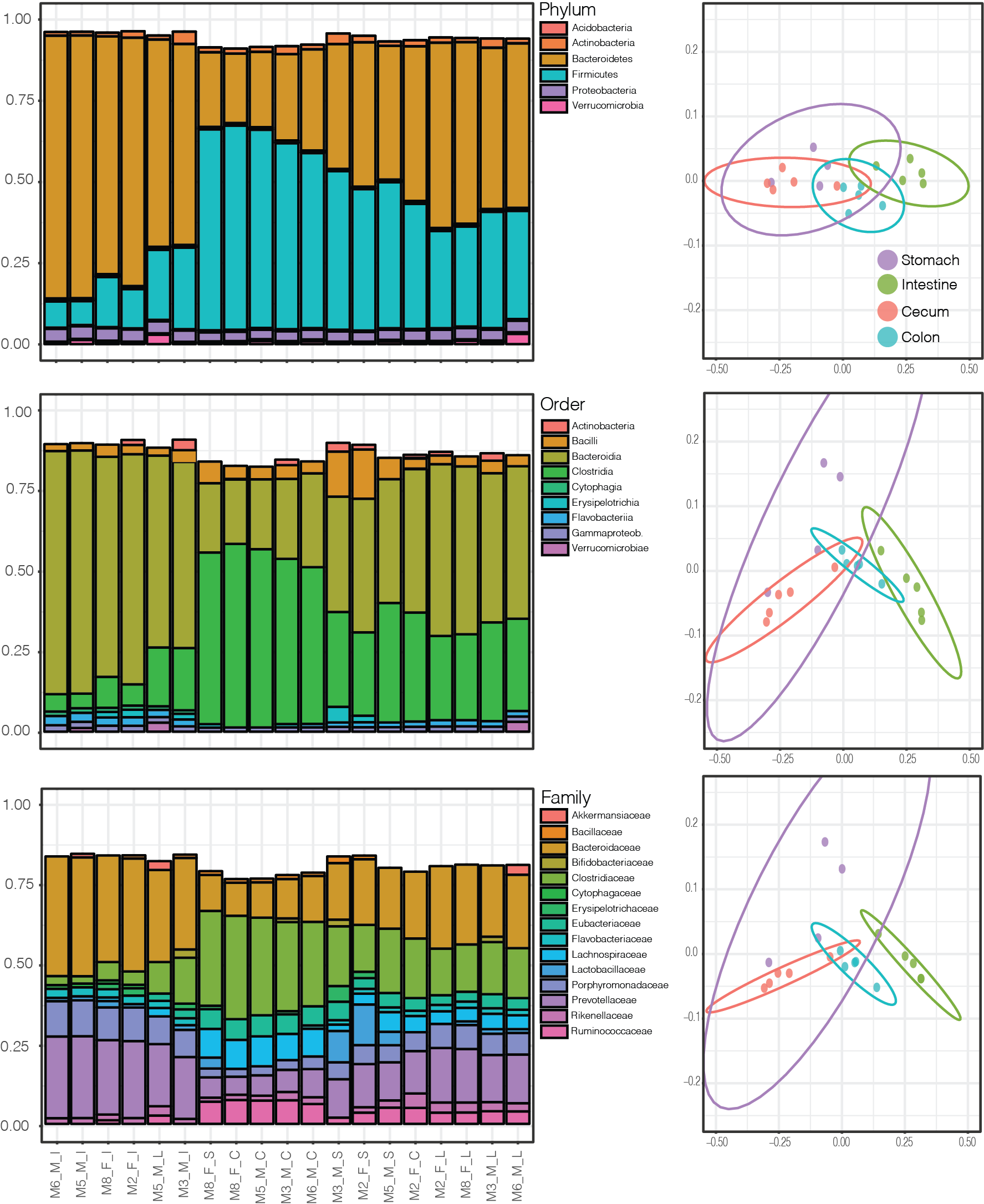

Supplement: Supplementary file 2 — Supplementary Figure 2. [file 41598_2020_63775_MOESM2_ESM.png]

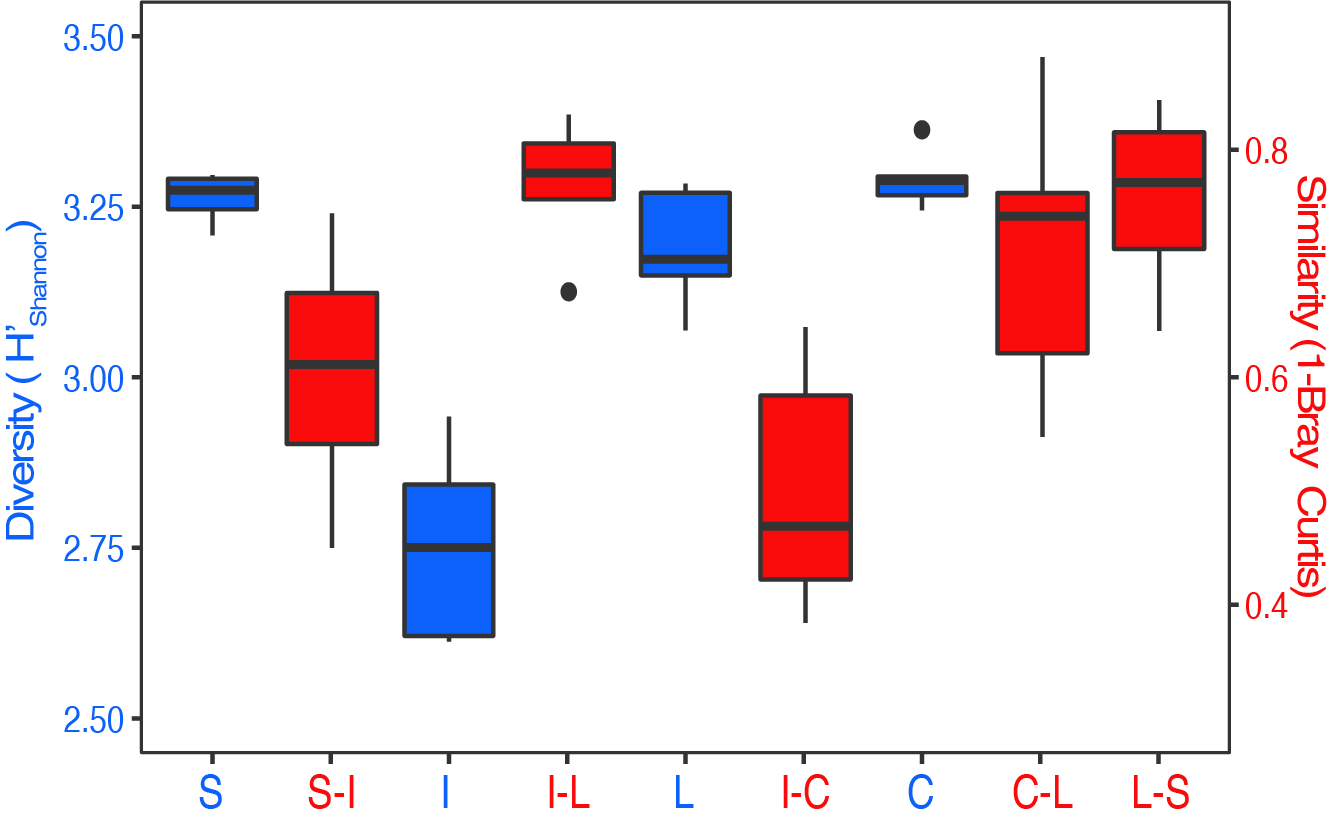

Supplement: Supplementary file 3 — Supplementary Figure 3. [file 41598_2020_63775_MOESM3_ESM.png]
